# Prevalence of Life Stressors and Posttraumatic Stress Disorder Among Women in Iceland

**DOI:** 10.1001/jamanetworkopen.2024.49430

**Published:** 2024-12-06

**Authors:** Edda Bjork Thordardottir, Huan Song, Qing Shen, Andri Bjornsson, Hildur Yr Hilmarsdottir, Berglind Guðmundsdottir, Engilbert Sigurdsson, Gunnar Tómasson, Harpa Rúnarsdóttir, Donghao Lu, Filip K. Arnberg, Jóhanna Jakobsdóttir, Fang Fang, Thor Aspelund, Arna Hauksdóttir, Unnur Anna Valdimarsdóttir

**Affiliations:** 1 Faculty of Medicine, School of Health Sciences, University of Iceland, Reykjavik, Iceland; 2 West China Biomedical Big Data Center, West China Hospital, Sichuan University, Chengdu, China; 3 Clinical Research Center for Mental Disorders, Shanghai Pudong New Area Mental Health Center, Tongji University School of Medicine, Shanghai, China; 4 Institute for Advanced Study, Tongji University, Shanghai, China; 5 Faculty of Psychology, School of Health Sciences, University of Iceland, Reykjavik, Iceland; 6 Mental Health Services, Landspitali - The National University Hospital of Iceland, Reykjavik, Iceland; 7 Instititute of Environmental Medicine, Karolinska Institutet, Stockholm Sweden; 8 Department of Medical Sciences, National Centre for Disaster Psychiatry, Uppsala University, Uppsala, Sweden; 9 Department of Epidemiology, Harvard T H Chan School of Public Health, Boston, Massachusetts

## Abstract

**Importance:**

Comprehensive data on the prevalence of various life stressors and their role in posttraumatic stress disorder (PTSD) among women are lacking.

**Objective:**

To determine the prevalence of a broad range of life stressors and their association with PTSD in a large nationally representative cohort of women.

**Design, Setting, and Participants:**

This cross-sectional analysis used data from the population-based Stress-And-Gene-Analysis, which invited women in Iceland to complete an online survey from March 1, 2018, to July 1, 2019. Participants were a nationally representative sample of women 18 to 69 years of age. This analysis was conducted from January 21, 2022, to September 13, 2024.

**Exposures:**

History of 23 life stressors assessed with the Life Events Checklist for the *Diagnostic and Statistical Manual of Mental Disorders* (Fifth Edition) (*DSM-5)* and additional survey questions.

**Main Outcomes and Measures:**

The main outcome was probable PTSD during the past month assessed for the worst life stressor experienced as evaluated with the PTSD Checklist for *DSM-5*. Modified Poisson log-linear models were used to assess prevalence ratios (PRs) of PTSD by type of life stressor, adjusting for multiple covariates, including number of life stressors and time since the worst life stressor.

**Results:**

Overall, 28 199 women participated in the study (mean [SD] age, 43.8 [13.7] years). The prevalence of life stressors varied substantially, ranging from 3.3% for war or armed conflict–related events to 66.4% for unwanted sexual experiences. The overall prevalence of probable PTSD was 15.9% and varied substantially across life stressor types. The highest prevalence of probable PTSD was for sexual assault (1427 of 3872 [36.9%]) and lowest for natural disasters (15 of 456 [3.3%]). Interpersonal trauma, specifically sexual assault (adjusted PR [APR], 6.66 [95% CI, 4.01-11.04]), other unwanted sexual experience (APR, 4.33 [95% CI, 2.53-7.42]), and physical assault (APR, 4.44 [95% CI, 2.63-7.49]) were associated with the highest prevalence increase of probable PTSD compared with natural disasters as the reference category.

**Conclusions and Relevance:**

This cross-sectional study of 28 199 women in Iceland, which ranks highest globally in gender equality, found that sexual and physical assaults were among the most common life stressors reported and were associated with the highest prevalence of probable PTSD. The propensity of such trauma to result in PTSD calls for widespread societal efforts to prevent sexual and physical violence and reduce gender-based disparities in mental health.

## Key Points

**Question:**

What are the most prevalent life stressors faced by women in a high-income Nordic country (Iceland), and which life stressors are associated with the highest prevalence of posttraumatic stress disorder (PTSD)?

**Findings:**

In this cross-sectional study of 28 199 women, lifetime exposure to sexual or physical assault was reported by 40% of women. Sexual and physical assaults were statistically significantly associated with the highest elevated prevalence of probable PTSD compared with natural disasters as the reference category.

**Meaning:**

These results suggest that widespread societal efforts are needed to prevent violence against women, and systematic screening of violence and PTSD in the health care system is warranted for targeted treatment and prevention of secondary health risks among women with PTSD.

## Teaser

This cross-sectional study investigates the prevalence of life stressors and their association with posttraumatic stress disorder in a nationally representative cohort of women from a high-income Nordic country (Iceland) with one of the highest gender-equality indices worldwide.

## Introduction

Posttraumatic stress disorder (PTSD) is characterized by a failure to recover from a severe stress reaction following exposure to trauma.^1^ PTSD can have detrimental consequences on the ability to function in daily life and is highly comorbid with chronic and fatal diseases^2,3^ and premature mortality.^4^ PTSD often remains undetected despite health care utilization being substantially higher among persons who live with the disorder.^5^

Globally, it is estimated that the majority of women have been exposed to trauma^4^ and that 3% to 16% will experience PTSD at some point during their life.^6,7,8,9^ The risk of PTSD varies by the type of trauma reported, with violence usually being associated with the highest risk.^10^ To date, few studies have assessed the prevalence of PTSD across a wide range of life stressors while adjusting for important confounders, such as the number of life stressors reported, a known risk factor for higher PTSD symptom burden,^10^ as well as for time since the worst life stressor. Further, having experienced an event that involves a threat to life, serious injury, or sexual violence is a prerequisite for PTSD symptoms.^1^ However, studies indicate that other events which are not currently classified as traumatic may led to PTSD, with implications for the estimation of the prevalence of PTSD.^11^

With this background, we leveraged data from the Stress-and-Gene-Analysis (SAGA) cohort, a nationwide population-based study of women in Iceland to examine the prevalence of a broad range of potentially traumatic events and probable PTSD. Iceland is a high-income Nordic country and is currently listed as having the highest gender-equality index worldwide based on health, education, economic, and political criteria.^12^ We further assessed the risk of probable PTSD across life stressors, controlling for sociodemographic variables, prior life stressors, and time since the worst life stressor.

## Methods

### Study Design and Population

Participants in this cross-sectional study were women in the SAGA cohort, a population-based study in Iceland specifically designed to assess the association of trauma with women’s health. Eligible individuals were women 18 to 69 years of age, Icelandic-speaking, residing in the country, and having an identifiable address or telephone number. Women signed an informed consent using electronic authentication before answering an online survey. Approvals for the study were granted from the National Bioethics Committee and the Icelandic Data Protection Authority. This study followed the Strengthening the Reporting of Observational Studies in Epidemiology (STROBE) reporting guideline.

From March 1, 2018, to July 1, 2019, a total of 104 197 women were invited to complete an online survey, of which 30 403 (29%) responded. A comparison of sociodemographic characteristics found that participants of the SAGA cohort represent the general Icelandic female population in terms of distribution of age, education level, geographical location, and monthly wages.^13^ For further information, see the study protocol in eAppendix 1 in Supplement 1.

### Measurement of Exposure: Life Stressors

Lifetime exposure to 23 stressors was obtained with the Life Events Checklist for the *Diagnostic and Statistical Manual of Mental Disorders* (Fifth Edition) (*DSM-5*) (LEC-5),^14^ which assesses a range of events known to result in PTSD, as well as additional survey questions. Both direct (ie, happened to me) and indirect (ie, witnessed it; happened to someone close to me) exposures were assessed. Participants could have experienced both direct and indirect exposures to the same type of life stressor. Additionally, participants were asked if they had been exposed to the following life stressors (response options, yes or no): stillbirth, miscarriage, abortion, having a child with a serious physical or mental disability, having a child taken away, bullying, divorce or break-up, adultery, rejection by spouse, or discrimination and humiliation (eAppendix 2 in Supplement 1).

Participants also received an open-ended question about exposure to other life stressors not stated in the questionnaire. In total, 10 521 women reported other life stressors, of which 3323 women chose one of these as their worst life stressor. One-third of the worst life stressors (1025 of 3323) were randomly selected, and a content analysis was conducted to gain insight into what these events were.

The following information was gathered for the worst life stressor: involved threat to life or sexual violence; was repeated (response options, yes or no); age at (first) exposure classified in years as younger than 12, 12 to 18, 19 to 39, and 40 or older). If the worst life stressor involved repeated exposure, the age when the first and last events occurred was reported. Time since the (most recent) event was classified in years as less than 5, 5 to 9, 10 to 19, 20 to 29, and 30 or more. Relation to the perpetrator(s) was assessed for interpersonal events (response options were 1 = relative and partner, and 2 = friend, acquaintance, coworker or other; with precedence to option 1).

### Measurement of Outcome: Probable PTSD

Participants who reported at least 1 life stressor answered the PTSD Checklist for *DSM-5 (*PCL-5)^15^ about the worst life stressor. The PCL-5 is a reliable and valid^16^ questionnaire assessing *DSM-5* criteria for PTSD during the past month, specifically it assesses re-experiencing (criterion B), avoidance (criterion C), negative alterations in cognition and mood (criterion D), and arousal (criterion E). Probable PTSD cases were defined as having a cutoff score of at least 33 on the PCL-5^17^ (scores ranged from 0 to 80, with higher scores indicating greater symptom severity) and meeting criterion A (exposure to actual or threatened death, serious injury, or sexual violence), B, C, D, and E (items rated ≥2 were defined as a symptom reported).^15^ To be sensitive to women’s experience, women reporting an abortion or miscarriage were not asked if the event involved threat to life (ie, criterion A). We therefore only assessed the prevalence of PTSD criterion B to E for these events.

### Covariates

The SAGA cohort survey included questions on demographic characteristics. Covariates included age, education level, employment status, monthly income, relationship status, and number of children (eAppendix 2 in Supplement 1). The number of life stressors was also included as a covariate, as it has been found to be a risk factor for subsequent trauma exposure.^10^

### Statistical Analysis

The Pearson χ^2^ test was performed to determine whether women with or without probable PTSD differed with respect to background characteristics. The prevalence of the worst life stressor was calculated as the proportion of participants who experienced a specific life stressor and reported that it currently bothered them most. The prevalence of probable PTSD for each life stressor was calculated as the proportion of participants whose worst life stressor met *DSM-5* criteria for PTSD.^1^

We used modified Poisson log-linear models for binary response data with robust error variance to obtain prevalence ratios (PRs) with 95% CIs of probable PTSD. Before applying the Poisson models, we demonstrated that the logistic regression models for the binary response were adequate according to the Hosmer-Lemeshow goodness of fit test.

The presented PRs represent a ratio of the proportion of participants with probable PTSD in a particular life stressor category (eg, after physical assault), with PTSD prevalence after natural disasters as a reference. Natural disasters are a common life stressor in Iceland and are associated with low PTSD prevalence in population-based samples.^18^ Similarly, we assessed the associations between worst life stressor categories and risk of PTSD, with or without criterion A, as prior studies have indicated that life stressors which do not met criterion A may result in probable PTSD.^11^ The PRs were adjusted for age, income, educational level, marital status, number of children, and number of life stressors. We also adjusted for years since the worst life stressor because close temporal proximity to that experience has been found to be associated with increased risk of PTSD.^19^

We analyzed the prevalence of probable PTSD using logistic regression models by types of life stressors. As assaults were among the most common types of life stressors reported by women in our study, we performed analyses by relationship to the perpetrator and whether the violence was repeated or not. Adjustments were made for current age, number of life stressors, and years since the worst life stressor occurred. Further analysis was performed to assess the potential association of age at (first) assault and time since (most recent) assault with PTSD prevalence among women reporting assaults as their worst life stressor using trend tests based on polynomial contrasts. Adjustments were made for current age and number of life stressors. Similarly, the prevalence of probable PTSD for 11 different life stressors was studied collectively across age at the (first) event and time since the (most recent) event to assess the possible moderating association of age and time. The model-based (marginal means) estimates are shown in eFigures 2 and 3 in Supplement 1. The significance of the moderating association was evaluated by incorporating a statistical interaction term in the models. This was done in one model for the life stressor type and age at exposure, and in another model for the life stressor type and time of the (first) event.

We row-imputed items for participants who responded to more than 75% of each subcluster on the PCL-5 and then calculated the total score. The PTSD analyses were conducted using imputed PCL-5 data. For comparison, the main analyses were repeated in the original dataset with participants having complete answers on the PCL-5 (eTable 1 in Supplement 1).

All analyses were conducted January 21, 2022, to September 13, 2024, with SAS software, version 9.4, (SAS Institute Inc) and R statistical software, version 4.4.1 (R Project for Statistical Computing). A 2-sided value of *P* < .05 was considered statistically significant.

## Results

Women with missing items on exposure to life stressors (n = 1949) and women with more than 25% missing items on the PCL-5 (n = 255) were excluded from the analysis, which resulted in a study population of 28 199 women. Among them, the mean (SD) age was 43.8 (13.7) years, 54.4% held a university degree, 73.6% were employed or studying, and 37.7% reported medium or high income. Most respondents were married or in a relationship (75.6%), had 1 or more child (80.4%), and reported having been directly exposed (“happened to me”) to a life stressor on the LEC-5 questionnaire (83.8%) (Table 1).

**Table 1.** Demographic Characteristics of the SAGA Cohort by Probable PTSD

Characteristic	Participants, No. (%)			*P* value
	Overall	Probable PTSD		
		No	Yes	
Overall	28 199 (100)	23 712 (84.1)	4487 (15.9)	
Age range, y				
18-29	5475 (19.4)	4239 (77.4)	1236 (22.6)	<.001
30-39	5769 (20.5)	4803 (83.3)	966 (16.7)	
40-49	6263 (22.2)	5320 (84.9)	943 (15.1)	
50-59	6392 (22.7)	5551 (86.8)	841 (13.2)	
60-69	4300 (15.3)	3799 (88.4)	501 (11.7)	
Educational level				
Primary school	4115 (14.7)	3013 (73.2)	1102 (26.8)	<.001
Secondary school	8700 (31.0)	7043 (81.0)	1657 (19.1)	
Tertiary A	8934 (31.8)	7823 (87.6)	1111 (12.4)	
Tertiary B	6338 (22.6)	5755 (90.8)	583 (9.2)	
Declined to answer	112	78	34	
Employment status				
Employed or studying	21 360 (73.6)	18 645 (87.3)	2715 (12.7)	<.001
Receiving disability benefits	2643 (9.4)	1727 (65.3)	916 (34.7)	
Retired or parental leave or staying at home	2487 (8.8)	2160 (86.9)	327 (13.2)	
Unemployed or on sick leave	1493 (5.3)	1031 (69.1)	462 (30.9)	
Declined to answer	216	149	67	
Income				
Low	8422 (31.1)	6352 (75.4)	2070 (24.6)	<.001
Low-medium	8454 (31.2)	7160 (84.7)	1294 (15.3)	
Medium	6446 (23.8)	5812 (90.2)	634 (9.8)	
Medium-high	2777 (10.3)	2538 (91.4)	239 (8.6)	
High	982 (3.6)	912 (92.9)	70 (7.1)	
Declined to answer	1118	938	180	
Relationship status				
Married or in a relationship	21 214 (75.6)	18 297(86.3)	2917 (12.8)	<.001
Single	6270 (22.4)	4850 (77.3)	1420 (22.7)	
Widowed	564 (2.0)	464 (82.3)	100 (17.7)	
Declined to answer	151	101	50	
No. of children				
None	5248 (19.6)	4214 (80.3)	1034 (19.7)	<.001
1-2	10 552 (39.3)	8995 (85.2)	1557 (14.8)	
3-4	9944 (37.1)	8533 (85.8)	1411 (14.2)	
≥5	1079 (4.0)	858 (79.5)	221 (20.5)	
Declined to answer	1376	1112	264	
No. of LEC-5 events (happened to me)				
0	4555 (16.2)	4415 (96.9)	140 (3.1)	<.001
1	6138 (21.8)	5693 (92.7)	445 (7.3)	
2	6301 (22.3)	5519 (87.6)	782 (12.4)	
3	4726 (16.8)	3782 (80.0)	944 (20.0)	
4	3233 (11.5)	2363 (73.1)	870 (26.9)	
≥5	3246 (11.5)	1940 (59.8)	1306 (40.2)	

Abbreviations: LEC-5, Life Events Checklist for *Diagnostic and Statistical Manual of Mental Disorders* (Fifth Edition); PTSD, posttraumatic stress disorder; SAGA, Stress-and-Gene-Analysis.

A total of 4487 (15.9%) women were classified as having probable PTSD based on their score on the PCL-5 questionnaire. Younger women were more likely to have probable PTSD than older women (1236 [22.6%] vs 501 [11.7%]; *P* < .001), and women with primary school education were more likely to have probable PTSD than women with B-level tertiary education (1102 [26.8%] vs 583 [9.2%]; *P* < .001). Women receiving disability benefits (916 [34.7%]) and women unemployed or on sick leave (462 [30.9%]) were also more likely to have probable PTSD than women who were employed or studying (2715 [12.7%]; *P* < .001). Probable PTSD was also more prevalent among women with low income compared with women with high income (2070 [24.6%] vs 70 [7.1%]; *P* < .001) and among single women compared with women in a relationship (1420 [22.7%] vs 2917 [12.8%]; *P* < .001). Moreover, women with no children (1034 [19.7%]) or more than 5 children (221 [20.5%]) were more likely to have PTSD compared with women with 1 to 2 children (1557 [14.8%]) or 3 to 4 children (1411 [14.2%]; *P* < .001). Reporting 5 or more events on the LEC-5 was further associated with probable PTSD (1306 [40.2%]) compared with reporting fewer events (445 [7.3%] for 1 event and 782 [12.4%] for 2 events; *P* < .001)

### Prevalence of Life Stressors

Among 28 199 participants, the most frequent life stressors reported as direct or indirect exposures were unwanted sexual experiences (66.4%), life-threatening illness or injury (58.1%), and fire, explosion, or accident (51.1%). The least common life stressor reported was exposure to war or to armed conflict (3.3%). When direct exposure (ie, happened to me) was assessed exclusively, the most frequently reported life stressors were divorce or break-up (62.3%), bullying (53.9%), and unwanted sexual experiences (56.9%) (Table 2). Being directly exposed to either a sexual or physical assault or both types of assaults was reported by 11 233 women (39.8%).

**Table 2.** Prevalence of Life Stressors and Worst Life Stressor Categories Among 28 199 Women in the SAGA Cohort

Life stressor	Participants, No. (%)			
	Direct or indirect exposure^a^	Happened to me	Worst life stressor^b^	Criterion A^c^
**LEC-5**				
Natural disaster	11 499 (40.8)	7563 (26.8)	456 (4.0)	244 (53.5)
Fire, explosion, or serious accident	14 401 (51.1)	6922 (24.6)	990 (6.9)	836 (84.4)
Life-threatening illness or injury	16 382 (58.1)	4906 (17.4)	3606 (22.0)	3606 (100)
War or armed conflict	920 (3.3)	206 (0.7)	24 (2.6)	21 (87.5)
Unwanted sexual experiences	18 718 (66.4)	16 054 (56.9)	5661 (30.2)	5661 (100)
Sexual assault	13 012 (46.1)	8630 (30.6)	3872 (29.8)	3872 (100)
Other unwanted sexual experience (exclusively)^d^	7424 (26.3)	5706 (20.2)	1789 (24.1)	1789 (100)
Captivity	1173 (4.2)	672 (2.4)	82 (7.0)	72 (87.8)
Physical assault	11 241 (39.9)	6235 (22.1)	1423 (12.7)	1056 (74.2)
Sudden violent death	7107 (25.2)	NA^e^	1143 (16.1)	1143 (100)
Sudden death due to accident	8051 (28.6)	NA^e^	1293 (16.1)	1293 (100)
Caused serious injury or harm to someone else	NA^e^	370 (1.3)	34 (9.9)	34 (100)
**Other life stressors**				
Abortion	NA^f^	6377 (22.6)	407 (6.4)	NA^g^
Miscarriage	NA^f^	8877 (31.5)	688 (7.8)	NA^g^
Stillbirth	NA^f^	792 (2.8)	265 (33.5)	265 (100)
Other difficult birth experience	NA^f^	8416 (29.9)	700 (8.3)	205 (29.3)
Child taken away	NA^f^	411 (1.5)	86 (20.9)	48 (55.8)
Child with serious disability	NA^f^	2360 (8.4)	601 (25.5)	373 (62.1)
Divorce or break-up	NA^f^	17 568 (62.3)	1978 (11.3)	428 (21.6)
Adultery or rejection by spouse	NA^f^	12 088 (42.9)	1661 (13.7)	274 (16.5)
Bullying	NA^f^	15 191 (53.9)	2197 (14.5)	395 (18.0)
Discrimination	NA^f^	2324 (8.2)	62 (2.7)	62 (100)
Humiliation	NA^f^	129 033 (45.8)	939 (7.3)	280 (29.8)
Other stressful life experience	10 436 (37.0)	NA	3290 (31.5)	1983 (60.3)

Abbreviations: LEC-5, Life Events Checklist for *Diagnostic and Statistical Manual of Mental Disorders* (Fifth Edition) (*DSM-5*); NA, not applicable; PTSD, posttraumatic stress disorder; SAGA, Stress-And-Gene-Analysis.

^a^ Happened to me, I witnessed it, or it happened to someone close to me.

^b^ Proportion of participants who chose the life stressor indicated in the column heading as their worst life stressor. For example, 4.0% of participants who experienced a natural disaster chose it as their worst life stressor.

^c^ Proportion of the worst life stressor that met the *DSM-5* PTSD diagnostic criterion A, that is, death, life threat, or sexual violence. For example, 53.5% of natural disaster experiences that were chosen as the worst life stressor met criterion A.

^d^ Does not include women who had experienced a sexual assault.

^e^ Does not apply.

^f^ No information available about indirect exposure.

^g^ No information available about whether the event met *DSM-5* PTSD diagnostic criterion A.

Among women endorsing a particular life stressor, the life stressors most frequently chosen as the worst were stillbirth (265 of 792 [33.5%]), other stressful life experiences (3290 of 10 436 [31.5%]), and unwanted sexual experiences (5661 of 18 718 [30.2%]) (Table 2). The least common worst life stressors were exposure to war or armed conflict (24 of 920 [2.6%]), discrimination (62 of 2324 [2.7%]), and natural disaster (456 of 11 499 [4.0%]). Of life stressors that do not by definition meet *DSM-5* criterion A for PTSD, those most likely to fulfill criterion A were captivity (72 of 82 [87.8%]), fire or explosion or serious accident (836 of 990 [84.4%]), and exposure to war or armed conflict (21 of 24 [87.5%]). The worst life stressor most unlikely to fulfill criterion A was adultery or rejection by spouse (274 of 1661 [16.5%]). The prevalence of life stressors by exposure type (ie, happened to me, witnessed it, and happened to someone close to me) is presented in eTable 2 in Supplement 1.

About one-third of participants (10 436 [37.0%]) reported experiencing other life stressors in an open-ended question on the LEC-5. Of them, 3290 (31.5%) chose this experience as their worst. A content analysis of 1025 of these events showed the most reported were related to mental illness (their own or others; 32.5%), difficult family situations (21.4%), psychological violence (13.8%), and death of a family member or friend (12.2%) (eFigure 1 in Supplement 1).

### Prevalence of Probable PTSD by Type of Life Stressor

Interpersonal trauma, specifically sexual assault (1427 of 3872 [36.9%]), and being held captive (27 of 82 [32.9%]) carried the highest prevalence of probable PTSD. Having a child taken away (26 of 86 [30.2%]), physical assault (373 of 1423 [26.2%]), and sudden violent death (215 of 1143 [18.8%]) were also associated with high prevalence of probable PTSD (Table 3). Direct exposure to natural disasters was relatively common (7563 of 28 199 [26.8%]) but associated with low prevalence of probable PTSD (15 of 456 [3.3%]), while being held captive (672 of 28 199 [2.4%]) and having a child taken away (411 of 28 199 [1.5%]) were less common but associated with a high prevalence of probable PTSD (27of 82 [32.9%] for being held captive and 26 of 86 [30.2%] for having a child taken away) (Table 2 and Table 3).

**Table 3.** Prevalence of Probable PTSD by Life Stressor Category

Life stressor	Probable PTSD No./total No. (%)^a^	Adjusted PR (95% CI)^b^	Further adjusted PR (95% CI)^c^
**LEC-5**			
Natural disaster	15/456 (3.3)	1.00 [Reference]	1.00 [Reference]
Fire, explosion, or serious accident	129/990 (12.0)	3.96 (2.32-6.74)	2.53 (1.48-4.34)
Life-threatening illness or injury	537/3606 (14.9)	4.29 (2.59-7.10)	2.66 (1.60-4.44)
War or armed conflict	<10	3.32 (1.10-9.99)	NA
Sexual assault	1427/3872 (36.9)	9.32 (5.65-15.39)	6.66 (4.01-11.04)
Other unwanted sexual experience (exclusively)^d^	298/1789 (16.7)	4.91 (2.93-8.23)	4.33 (2.53-7.42)
Captivity	27/82 (32.9)	7.17 (4.03-12.74)	NA
Physical assault	373/1423 (26.2)	7.57 (4.55-12.60)	4.44 (2.63-7.49)
Sudden violent death	215/1143 (18.8)	4.99 (2.99-8.35)	3.12 (1.84-5.29)
Sudden death due to accident	179/1293 (13.8)	3.95 (2.36-6.61)	2.36 (1.40-3.97)
Caused serious injury or harm to someone else	<10	7.73 (3.23-18.54)	NA
**Other life stressors**			
Stillbirth	33/265 (12.5)	3.35 (1.87-5.97)	3.02 (1.70-5.33)
Other difficult birth experience	24/700 (3.4)	0.80 (0.44-1.47)	0.90 (0.46-1.77)
Child taken away	26/86 (30.2)	9.03 (4.91-16.63)	NA
Child with serious disability	84/601 (14.0)	4.09 (2.40-6.98)	3.50 (2.00-6.12)
Divorce or break-up	146/1978 (7.4)	2.18 (1.29-3.70)	1.42 (0.84-2.40)
Adultery or rejection by spouse	99/1661 (5.9)	1.77 (1.03-3.06)	1.21 (0.70-2.07)
Bullying	182/2197 (8.3)	2.32 (1.36-3.94)	2.05 (1.20-3.51)
Discrimination	<10	1.32 (0.36-4.87)	NA
Humiliation	125/939 (13.3)	3.90 (2.29-6.63)	2.69 (1.53-4.72)
Other stressful life experience	554/3290 (16.8)	4.76 (2.87-7.88)	2.87 (1.71-4.81)

Abbreviations: LEC-5, Life Events Checklist for *Diagnostic and Statistical Manual of Mental Disorders* (Fifth Edition); NA, not applicable; PR, prevalence ratio; PTSD, posttraumatic stress disorder.

^a^ The prevalence of probable PTSD by the life stressor type indicated in the column heading.

^b^ Adjusted for current age.

^c^ Adjusted for current age, income, education level, marital status, number of children, number of life stressors, and years since worst life stressor occurred.

^d^ Does not include women who reported a sexual assault.

When adjusting for all covariates, events involving interpersonal assault or threat were associated with the highest prevalence of probable PTSD (Table 3). Compared with exposure to natural disasters, we found that sexual assault (adjusted PR [APR], 6.66 [95% CI, 4.01-11.04]), and physical assault (APR, 4.44 [95% CI, 2.63-7.49]) were associated with the highest overall elevated prevalence of probable PTSD. Using the same reference group, other unwanted sexual experiences (APR, 4.33 [95% CI, 2.53-7.42]) and witnessing a sudden violent death (APR, 3.12 [95% CI, 1.84-5.29]) were also associated with considerably elevated prevalence of probable PTSD. Life stressors involving women’s children were associated with considerably elevated prevalence of probable PTSD, including having a child with a serious disability (APR, 3.50 [95% CI, 2.00-6.12]), and experiencing a stillbirth (APR, 3.02 [95% CI, 1.70-5.33]).

The prevalence of probable PTSD after divorce or break-up, adultery or rejection, or other difficult birth experience was not statistically significantly elevated as compared with natural disasters (Table 3). When releasing PTSD criterion A, however, these events became statistically significantly associated with elevated PTSD prevalence (Figure; eTable 3 in Supplement 1).

**Figure.**
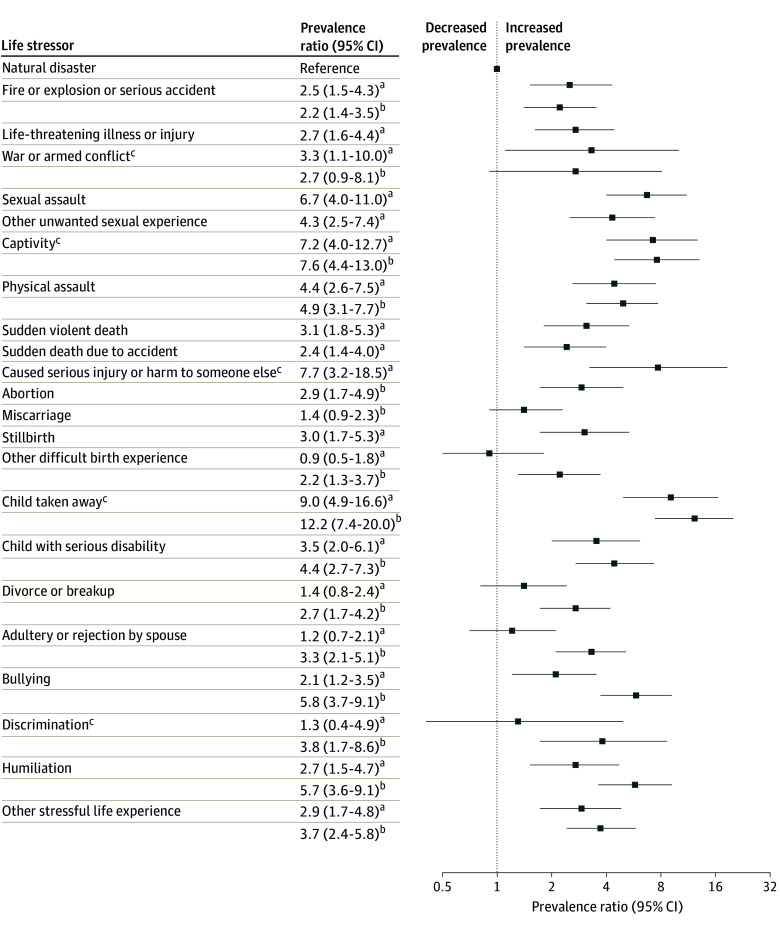
Association Between Probable Posttraumatic Stress Disorder (PTSD) and Life Stressor Categories With or Without *Diagnostic and Statistical Manual of Mental Disorders* (Fifth Edition) PTSD Diagnostic Criterion A Adjustments were made for current age, income, education level, marital status, number of children, number of life stressors, and years since the worst life stressor occurred. ^a^PTSD diagnostic criteria A through E met. ^b^PTSD diagnostic criteria B through E met (ie, without criterion A). ^c^Adjustments were made only for current age.

We also found a difference in probable PTSD prevalence across life stressors by age at the (first) event. For instance, for sexual assaults, the prevalence of probable PTSD was highest among women first assaulted when they were younger than 12 years of age (34.0% [95% CI, 31.5%-36.5%]) compared with women first assaulted when they were 19 to 39 years of age (23.9% [95% CI, 21.2%-26.7%]; *P* < .001). By contrast, the prevalence of probable PTSD for serious accidents was highest among women whose most recent accident was in older adulthood (≥40 years; 24.9% [95% CI, 18.4%-32.8%]) compared with in childhood (<12 years of age; 6.6% [95% CI, 3.5%-11.9%]; *P* < .001). For natural disasters and other stressful life experiences, the prevalence of probable PTSD was similar across age categories (eFigure 2 in Supplement 1).

Probable PTSD prevalence of time since the (most recent) event was similar across life stressors, with the moderating association of time being nonsignificant (eFigure 3 in Supplement 1). Generally, the prevalence of probable PTSD decreased as a function of duration of time from the (most recent) event across different life stressors (eFigure 3 in Supplement 1).

### Risk Factors of Probable PTSD After Sexual or Physical Assault

We found a curvilinear trend in the prevalence of probable PTSD by age at the (first) assault, with 723 of 1892 women (38.2%) assaulted before the age of 12 years, and 48 of 175 women (27.4%) assaulted at 40 years of age or older meeting probable PTSD criteria (test for curvilinear trend, *P* = .01). The prevalence of probable PTSD decreased as a function of duration of time from the (most recent) assault, with 577 of 1151 women (50.1%) who were assaulted 5 or less years ago meeting criteria for probable PTSD compared with 305 of 1242 women (24.6%) last assaulted 30 or more years ago (*P* < .001 for test of curvilinear trend). Assaults by a partner or relative were associated with a higher prevalence of probable PTSD than assaults by a friend, coworker, acquaintance or other (1000 of 2740 [36.5%] vs 800 of 2555 [31.3%]; *P* < .001). Repeated assault (vs 1 assault) was also associated with an increased prevalence of probable PTSD (1432 of 3672 [39.0%] vs 354 of 1525 [23.2%]; *P* < .001), particularly physical assaults (Table 4). When PTSD diagnostic criterion A was excluded, we found minimal changes in the associations between characteristics of physical assaults and probable PTSD (eTable 4 in Supplement 1).

**Table 4.** Prevalence of Probable PTSD by Characteristics of Sexual and Physical Assault Among Participants Reporting Assaults as Their Worst Life Stressor

Characteristic	Probable PTSD among participants, No./total No. (%)^a^		
	Sexual or physical assault	Sexual assault	Physical assault
Age at (first) assault, y^b^			
≥40	48/175 (27.4)	20/75 (26.7)	28/100 (28.0)
19-39	418/1403 (29.8)	261/844 (30.9)	157/559 (28.1)
12-18	597/1727 (34.6)	531/1441 (36.9)	66/286 (23.1)
<12	723/1892 (38.2)	602/1430 (42.1)	121/462 (26.2)
Test for linear trend^b^	*P* = .46	*P* = .17	*P* = .09
Test for curvilinear trend^b^	*P* = .01	*P* = .05	*P* = .09
Time since (most recent) assault, y^b^			
≥30	305/1242 (24.6)	260/963 (27.0)	45/229 (16.1)
20-29	250/877 (28.5)	196/626 (31.3)	54/251 (21.5)
10-19	367/1151 (31.9)	286/815 (35.1)	81/336 (24.1)
5-9	286/773 (37.0)	221/565 (39.1)	65/208 (31.3)
<5	577/1151 (50.1)	450/818 (55.0)	127/333 (38.1)
Test for linear trend^b^	*P* < .001	*P* < .001	*P* < .001
Test for curvilinear trend^b^	*P* < .001	*P* < .001	*P* = .94
Perpetrator^c^			
Partner or relative	1000/2740 (36.5)	684/1632 (41.9)	316/1108 (28.5)
Other^d^	800/2555 (31.3)	743/2240 (33.2)	57/315 (18.1)
Test for difference^c^	*P* < .001	*P* < .001	*P* < .001
Repeated assault^c^			
No	354/1525 (23.2)	313/1211 (25.9)	41/314 (13.1)
Yes	1432/3672 (39.0)	1101/2579 (42.7)	331/1093 (30.3)
Test for difference^c^	*P* < .001	*P* < .001	*P* < .001

Abbreviation: PTSD, posttraumatic stress disorder.

^a^ The prevalence of PTSD associated with characteristics of the assault indicated in the column heading.

^b^ Adjusted for current age and number of life stressors.

^c^ Adjusted for current age, number of life stressors, and years since the worst life stressor (the assault) occurred.

^d^ Friend, coworker, acquaintance, or other.

## Discussion

To our knowledge, this cross-sectional study is the largest nationwide population-based assessment of the prevalence of life stressors and corresponding associations with probable PTSD among women. The results of the study, comprising 28 199 women residing in a high-income Nordic country, indicated that assaults were among the most prevalent life stressors, with approximately 40% of the female population reporting lifetime occurrence of sexual or physical assaults. Assaults were also associated with the highest prevalence of probable PTSD, particularly during the first years after exposure, after repeated exposure, and in cases where the perpetrators were a partner or a relative.

In total, 15.9% of women in our study reported probable PTSD in the past month. Our estimated prevalence of probable PTSD is higher than previously reported, with the point prevalence of PTSD among women in high-income European countries estimated to be 1% to 7%,^7,8,9,20,21^ with the majority in the lower range. Similar estimates have been reported in high-income countries in North America and Australia.^22^ A plausible explanation for the higher prevalence of probable PTSD in our study is the use of the PCL-5 screening instrument rather than clinical diagnostic interviews as well as the broad range of potentially traumatic events addressed. Releasing the requirement for life stressors to involve life threat, serious injury, or sexual violence resulted in elevated prevalence of probable PTSD after several (noncriterion A) life stressors. In line with our results, prior studies have reported PTSD symptoms after noncriterion A stressors, such as bullying^23^ and childbirth.^24^ Notably, psychological violence was one of the most reported events in our content analysis of women’s worst life stressor in the category of “other stressful life experiences” and has previously been reported to result in PTSD after controlling for other types of violence.^25^ Thus, our findings have clear implications for the ongoing theoretical and evidence-based discussion of which life events can cause PTSD.^11^

The high lifetime prevalence of sexual or physical assault in this study is similar to estimates from Finland^26^ and Denmark^27^ (43% and 50% after age 15, respectively) and reports from Sweden (46% physically assaulted and 20% sexually assaulted).^28^ Adjusting for multiple covariates, sexual and physical assaults were among the life stressors associated with the highest prevalence of probable PTSD, which is similar to findings from the World Mental Health Surveys.^10^ In line with prior research,^19^ risk factors for probable PTSD after assaults were having a perpetrator who was a partner or relative, repeated assaults, and close temporal proximity to the assault. Nevertheless, we found a substantial proportion of women who experienced PTSD decades after the last assault, lending support to the chronicity of PTSD after exposure to such trauma.^10^

We found age at trauma exposure to have varying moderating associations with probable PTSD, depending on the trauma type. Our results suggested that childhood may be a sensitive period for developing PTSD after unwanted sexual experiences, while in contrast, the risk of PTSD following noninterpersonal trauma appears either consistent across the life span or increased in late adulthood. Previous studies indicate that younger age at trauma onset is associated with greater risk of PTSD, although research on this topic is limited.^22^ Moreover, consistent with previous studies, we found that close temporal proximity to the traumatic event is associated with elevated PTSD prevalence.^22^

Our findings add to the current understanding of PTSD among women by assessing a broader range of potentially traumatic events than previous studies have done. Stillbirths were, for instance, most likely to be reported as the worst event of all life stressors. A review of PTSD after prenatal loss found PTSD prevalence to range from 3% to 28% among mothers, highest 1 year after the loss.^29^ PTSD remains relatively unrecognized in the aftermath of stillbirth, with studies mainly conducted in clinical populations, highlighting the need for further studies.

### Limitations

Given the cross-sectional design of our study, the main limitation of this study pertains to potential recall bias in the assessment of life stressors. However, to explain the pattern in our results, this bias would need to be systematic across PTSD criteria and varying life stressors. We have no data to support that claim; to the contrary, the pattern in our results aligns well with patterns reported in previous studies.^10^ Our assessment of discrimination and humiliation was limited as we lacked information about the specific type of event women were exposed to. While we adjusted our analysis for potential confounding factors, including socioeconomic status and relationship status, it is possible that they are in some cases, at least partially, consequences of PTSD. Although our study sample was nationally representative of women 18 to 69 years of age in Iceland, our estimated prevalence of life stressors and probable PTSD was limited as 29% of Icelandic women participated in the study. We therefore cannot exclude the possibility of selection bias, which may have impacted the estimation of life stressors and PTSD prevalence in both directions, that is, toward lower prevalence if the most trauma-exposed were frail women who were unable to participate, or toward higher prevalence if women who have been exposed to life stressors were more likely to participate in the study than other women. The prevalence of life stressors in our study is, though, similar to previous Icelandic and Nordic studies.^30^ The generalizability of our results is limited to high-income countries scoring high on gender equality.

## Conclusions

In this cross-sectional study of 28 199 women residing in a Nordic welfare state with the highest gender-equality index worldwide, we found that 40% of women have been exposed to sexual or physical assaults and that these assaults were associated with the highest conditional risk of probable PTSD of all trauma types. These findings call for widespread societal efforts to prevent violence against women and systematic efforts to reduce gender-based disparities in mental health. This can be achieved through systematic screening of violence exposure and PTSD symptoms in health and social services and by increasing access to evidence-based treatments for PTSD.

## Notes

Supplement 1. **eAppendix 1.** Study protocol **eAppendix 2.** Exposure variables and covariates **eFigure 1.** The frequency of the most common life stressors that participants disclosed as their worst experience in an open-ended question on “other stressful life experiences” in the Life Events Checklist for *DSM-5* (LEC-5) **eFigure 2.** Prevalence of probable PTSD for life stressors by age at (first) event occurrence, expressed as marginal means estimates with 95% confidence intervals **eFigure 3.** Prevalence of probable PTSD for life stressors by time since (most recent) event, expressed as marginal means estimates and 95% confidence intervals **eTable 1.** Prevalence of probable PTSD by life stressor categories among participants with complete answers on the PCL-5 **eTable 2.** Prevalence of life stressors by exposure type in the study population (n = 28 199) **eTable 3.** Prevalence of probable PTSD without criterion A by life stressor category **eTable 4.** Prevalence of probable PTSD without criterion A by characteristics of physical assault among participants reporting physical assault as their worst life stressor

Supplement 2.
Data Sharing Statement
