# Supplementary material for: Prevalence of Life Stressors and Posttraumatic Stress Disorder Among Women in Iceland
Source: JAMA Netw Open. 2024 Dec 6;7(12):e2449430. doi: 10.1001/jamanetworkopen.2024.49430 (PMC11624579; doi:10.1001/jamanetworkopen.2024.49430)
Supplement: Supplement 1. — eAppendix 1. Study protocol eAppendix 2. Exposure variables and covariates eFigure 1. The frequency of the most common life stressors that participants disclosed as their worst experience in an open-ended question on “other stressful life experiences” in the Life Events Checklist for DSM-5 (LEC-5) eFigure 2. Prevalence of probable PTSD for life stressors by age at (first) event occurrence, expressed as marginal means estimates with 95% confidence intervals eFigure 3. Prevalence of probable PTSD for life stressors by time since (most recent) event, expressed as marginal means estimates and 95% confidence intervals eTable 1. Prevalence of probable PTSD by life stressor categories among participants with complete answers on the PCL-5 eTable 2. Prevalence of life stressors by exposure type in the study population (n = 28 199) eTable 3. Prevalence of probable PTSD without criterion A by life stressor category eTable 4. Prevalence of probable PTSD without criterion A by characteristics of physical assault among participants reporting physical assault as their worst life stressor [file jamanetwopen-e2449430-s001.pdf]

Supplemental Online Content

Thordardottir EB, Song H, Shen Q, et al. Prevalence of life stressors and posttraumatic stress disorder among women in Iceland. *JAMA Netw Open*. 2024;7(12):e2449430. doi:10.1001/jamanetworkopen.2024.49430

eAppendix 1. Study protocol

eAppendix 2. Exposure variables and covariates

eFigure 1. The frequency of the most common life stressors that participants disclosed as their worst experience in an open-ended question on “other stressful life experiences” in the Life Events Checklist for DSM-5 (LEC-5)

eFigure 2. Prevalence of probable PTSD for life stressors by age at (first) event occurrence, expressed as marginal means estimates with 95% confidence intervals

eFigure 3. Prevalence of probable PTSD for life stressors by time since (most recent) event, expressed as marginal means estimates and 95% confidence intervals

eTable 1. Prevalence of probable PTSD by life stressor category among participants with complete answers on the PCL-5

eTable 2. Prevalence of life stressors by exposure type in the study population (n=28199)

eTable 3. Prevalence of probable PTSD without criterion A by life stressor category

eTable 4. Prevalence of probable PTSD without criterion A by characteristics of physical assault among participants reporting physical assault as their worst life stressor

This supplemental material has been provided by the authors to give readers additional information about their work.

eAppendix 1. STUDY PROTOCOL

Proposal for the SAGA cohort

Title of the project: Significant life stressors and PTSD among women in Iceland: a nationwide cohort study

Investigators (responsible and others, including students):
Edda Bjork Thordardottir
Hildur Yr Hilmarsdottir
Arna Hauksdottir
Unnur Valdimarsdottir
Andri Steinthor Bjornsson

SAGA sponsor/investigator: Edda Björk Þórðardóttir

Background/rationale (2-4 sentences with references): The majority of women experience significant life stressors during their lifetime, such as exposure to violence, accidents, or being diagnosed with a life-threatening illness. Research on significant life stressors and symptoms of posttraumatic stress (PTSD) among women in Iceland is scarce, with the majority of knowledge based on a limited number of small studies.

Specific aims: The aim of this study is to assess the prevalence of significant life stressors and symptoms of PTSD among the Icelandic female adult population as well as the association of background characteristics (e.g. age and socioeconomic status) and PTSD symptoms. In addition, to assess the prevalence of general anxiety and association of 1) trauma history and 2) background characteristics and anxiety symptoms.

Methods: Descriptive analyses will be conducted to examine individual characteristics, history of exposure to significant life stressors and PTSD symptoms. We will use Poisson log-linear models with robust error variance to obtain relative risks (RRs) with 95% confidence intervals (CIs) of PTSD. All models will be adjusted for age (continuous).

Variables requested (see a detailed list in excel sheet):

a. **Exposure variables:** Life Events Checklist for DSM-5; social trauma; other potentially significant life stressors; age; education; finances; current living situation; employment status; number of children.
b. **Outcome variables:** Posttraumatic Stress Disorder Checklist for DSM-5; GAD-7
c. **Covariates*:** age

© 2024 Thordardottir EB et al. *JAMA Network Open.*

eAppendix 2. EXPOSURE VARIABLES AND COVARIATES

Exposure variables

In addition to the Life Events Checklist for DSM-5 (LEC-5), information about the following life stressors was assessed (response options yes/no):

1. Gestation and birth-related events, including stillbirth, miscarriage, abortion
2. Having a child with a serious physical or mental disability (e.g., impaired development, birth defect, impaired vision or hearing or walking abnormalities)
3. Having a child taken away from you
4. Bullying, defined as repeated reprehensible behavior of one person or many, intended to frighten, hurt, or intimidate others
5. Divorce or break-up
6. Adultery or rejection by spouse
7. Discrimination, such as prejudice on the grounds of race or sexual orientation
8. Humiliation (comprising: 1) being humiliated online/in media; 2) other humiliating experience)

Covariates

1. Age (divided into five groups for descriptive purposes: 18–29, 30-39, 40–49, 50–59, or 60–69 years; used as a continuous variable in all models)
2. Education level (primary, secondary, tertiary A, and tertiary B)
3. Current employment (employed, student, on disability pension, unemployed or on sick leave, retired, homemaker, or on parental leave)
4. Current monthly income (low (≤$2527), low-medium ($2528-$4212), medium ($4213-$5897), medium-high ($5898-$8424), and high (>$8425; conversion rates according to Central Bank of Iceland, October 17, 2018)
5. Relationship status (married or in a relationship, single, or widowed)
6. Number of children
7. Number of life stressors

© 2024 Thordardottir EB et al. *JAMA Network Open.*

SUPPLEMENTARY FIGURES

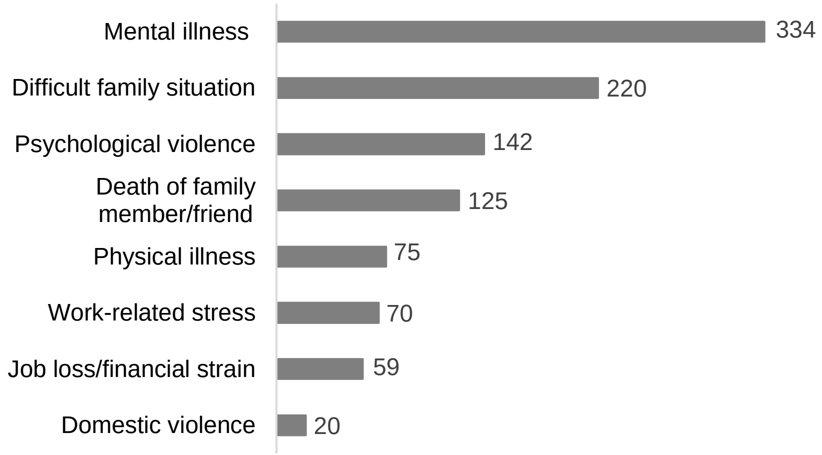

eFigure 1. The frequency of the most common life stressors that participants disclosed as their worst experience in an open-ended question on "other stressful life experiences" in the Life Events Checklist for DSM-5 (LEC-5)

© 2024 Thordardottir EB et al. *JAMA Network Open.*

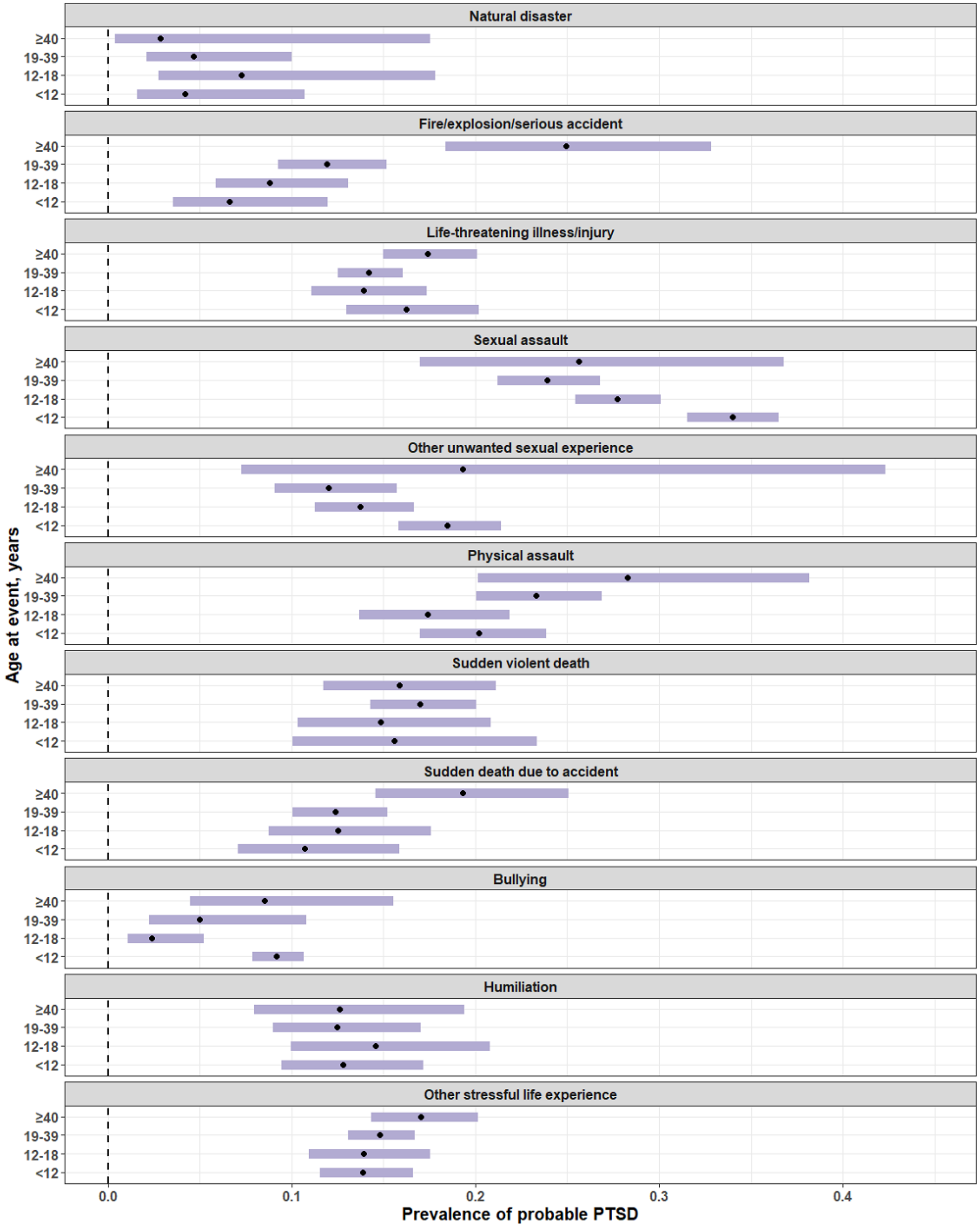

eFigure 2. Prevalence of probable PTSD for life stressors by age at (first) event occurrence, expressed as marginal means estimates with 95% confidence intervals

© 2024 Thordardottir EB et al. *JAMA Network Open.*

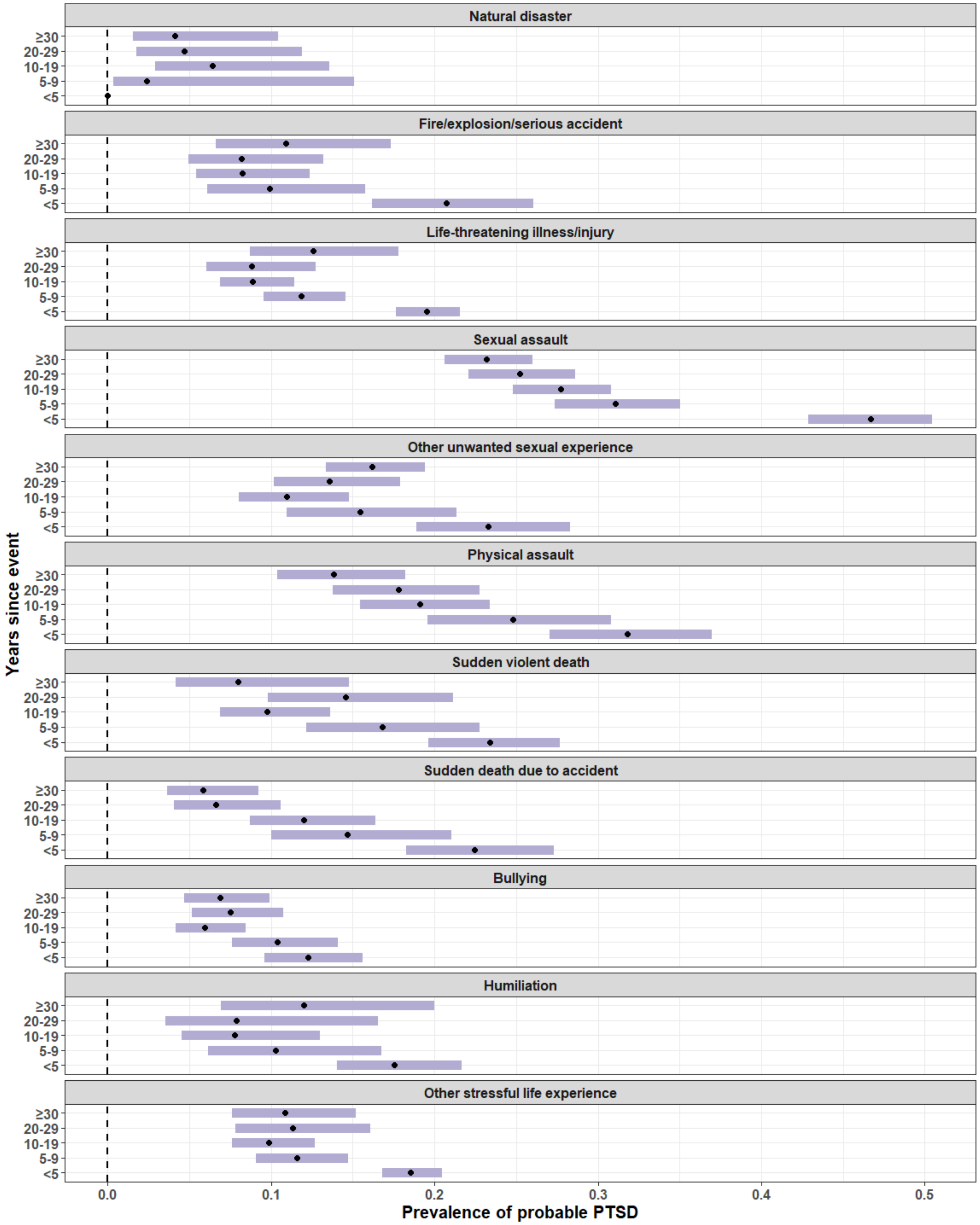

eFigure 3. Prevalence of probable PTSD for life stressors by time since (most recent) event, expressed as marginal means estimates and 95% confidence intervals

© 2024 Thordardottir EB et al. *JAMA Network Open.*

SUPPLEMENTARY TABLES

eTable 1. Prevalence of probable PTSD by life stressor category among participants with complete answers on the PCL-5

Life stressor	Probable PTSD No./total No. (%)[a]	PR (95% CI)[b]	PR (95% CI)[c]
**Life Events Checklist for DSM-5**			
Natural disaster	14/435 (3.2)	1.00 (ref)	1.00 (ref)
Fire/explosion/ serious accident	117/923 (12.7)	3.83 (2.20-6.65)	2.37 (1.36-4.13)
Life-threatening illness/injury	484/3389 (14.4)	4.19 (2.49-7.07)	2.54 (1.50-4.31)
War/ armed conflict	N/A	3.49 (1.15-10.58)	N/A
Sexual assault	1284/3528 (36.4)	9.23 (5.50-15.51)	6.64 (3.93-11.23)
Other unwanted sexual experience	259/1632 (15.9)	4.82 (2.82-8.24)	4.38 (2.49-7.68)
Captivity	27/77 (35.1)	7.79 (4.29-14.18)	N/A
Physical assault	342/1293 (26.5)	7.70 (4.54-13.05)	4.54 (2.65-7.81)
Sudden violent death	196/1060 (18.5)	4.95 (2.90-8.42)	3.23 (1.87-5.57)
Sudden death due to accident	159/1200 (13.3)	3.83 (2.25-6.54)	2.24 (1.31-3.83)
Caused serious injury/ harm to someone else	7/32 (21.9)	7.00 (2.74-17.83)	N/A
**Other life stressors**			
Stillbirth	30/256 (11.7)	3.21 (1.76-5.86)	3.18 (1.79-5.64)
Other difficult birth experience	23/671 (3.4)	0.82 (0.44-1.53)	0.95 (0.47-1.89)
Child taken away	22/79 (27.9)	8.14 (4.30-15.43)	N/A
Child with serious disability	72/545 (13.2)	3.96 (2.27-6.92)	3.18 (1.79-5.64)
Divorce/break-up	133/1861 (7.2)	2.15 (1.25-3.72)	1.42 (0.83-2.42)
Adultry/rejection by spouse	90/1561 (5.8)	1.74 (0.99-3.07)	1.16 (0.66-2.03)
Bullying	165/2012 (8.2)	2.31 (1.33-4.01)	2.19 (1.24-3.84)
Discrimination	n<10	1.38 (0.37-5.11)	N/A
Humiliation	118/850 (13.9)	4.13 (2.38-7.17)	2.85 (1.60-5.11)
Other stressful life experience	512/3010 (17.0)	4.92 (2.92-8.30)	2.92 (1.71-4.99)

[a] The prevalence of PTSD associated with the life stressor type indicated in the column heading.
[b] Adjusted for current age.
[c] Adjusted for current age, income, education level, marital status, number of children, number of life stressors and years since worst life stressor occurred.

© 2024 Thordardottir EB et al. *JAMA Network Open.*

eTable 2. Prevalence of life stressors by exposure type in the study population (n=28199)[a]

Life stressor	Overall[b] No. (%)	Happened to me No. (%)	Witnessed No. (%)	Happened to someone close to me No. (%)
Natural disasters	11499 (40.8)	7563 (26.8)	2434 (8.6)	3346 (11.9)
Fire/explosion/serious accident	14401 (51.1)	6922 (24.6)	2798 (9.9)	7493 (26.5)
Life-threatening illness/injury	16382 (58.1)	4906 (17.4)	2326 (8.2)	12045 (42.7)
War/armed conflict	920 (3.3)	206 (0.7)	160 (0.6)	608 (2.1)
Sexual assault	13012 (46.1)	8630 (30.6)	425 (1.5)	6468 (22.9)
Other unwanted sexual experience	16777 (59.4)	14782 (52.4)	1195 (4.2)	4773 (16.9)
Captivity	1173 (4.2)	672 (2.4)	128 (0.5)	456 (1.6)
Physical assault	11241 (39.8)	6235 (22.1)	3363 (11.9)	4800 (17.0)
Sudden violent death	7107 (25.2)	N/A[c]	645 (2.3)	6660 (23.6)
Sudden death due to accident	8051 (28.6)	N/A[c]	1038 (3.7)	7267 (29.5)

[a] Participants could endorse multiple levels of exposure to the same life stressor.
[b] Happened to me, witnessed it and/or happened to someone close to me.
[c] Does not apply.

eTable 3. Prevalence of probable PTSD without criterion A by life stressor category

Life stressor	Probable PTSD without criterion A No./total No. (%)[a]	PR (95% CI)[b]	PR (95% CI)[c]
**Life Events Checklist for DSM-5**			
Natural disaster	19/456 (4.2)	1.00 (ref)	1.00 (ref)
Fire/explosion/ serious accident	136/990 (13.7)	3.31 (2.06-5.32)	2.19 (1.36-3.54)
War/armed conflict	N/A	2.72 (0.92-8.09)	N/A
Captivity	32/82 (39.0)	7.59 (4.43-13.02)	5.39 (2.67-10.91)
Physical assault	460/1423 (32.3)	7.47 (4.77-11.71)	4.89 (3.10-7.72)
**Other life stressors**			
Other difficult birth experience	66/700 (9.4)	1.78 (1.09-2.90)	2.16 (1.26-3.72)
Child taken away	44/86 (51.2)	12.16 (7.41-19.95)	8.64 (4.72-15.82)
Child with serious disability	122/601 (20.3)	4.72 (2.96-7.54)	4.41 (2.66-7.31)
Divorce/break-up	388/1978 (19.6)	4.42 (2.82-6.94)	2.71 (1.73-4.24)
Adultry/rejection by spouse	411/1661 (24.7)	5.45 (3.48-8.54)	3.27 (2.09-5.11)
Bullying	561/2197 (25.5)	6.27 (3.99-9.84)	5.80 (3.70-9.08)
Discrimination	10/62 (16.1)	3.81 (1.69-8.58)	N/A
Humiliation	324/939 (34.5)	8.16 (5.19-12.82)	5.72 (3.62-9.05)
Abortion	48/407 (11.8)	2.38 (1.43-3.98)	2.86 (1.68-4.86)
Miscarriage	60/688 (8.72)	1.59 (0.98-2.56)	1.41 (0.87-2.29)
Other stressful life experience	880/3290 (26.8)	6.12 (3.92-9.54)	3.69 (2.35-5.79)

[a] The prevalence of probable PTSD (without criterion A) associated with the life stressor type indicated in the column heading.
[b] Adjusted for current age.
[c] Adjusted for current age, income, education level, relationship status, number of children, number of life stressors and years since the worst life stressor occurred.

© 2024 Thordardottir EB et al. *JAMA Network Open.*

eTable 4. Prevalence of probable PTSD without criterion A by characteristics of physical assault among participants reporting physical assault as their worst life stressor

Characteristics	Probable PTSD without criterion A No./total No. (%)[a]
**Age at (first) assault, y[b]**	
≥ 40	36/92 (39.1)
19-39	164/518 (31.7)
12-18	68/260 (26.3)
<12	143/412 (34.7)
Test for linear trend [b]	P=0.02
Test for curvilinear trend [b]	P=0.002
**Time since (most recent) assault, y[b]**	
≥ 30	53/243 (21.8)
20-29	55/228 (24.1)
10-19	90/315 (28.6)
5-9	73/193 (37.8)
<5	140/303 (46.2)
Test for linear trend [b]	P<0.001
Test for curvilinear trend [b]	P=0.254
**Perpetrator[c]**	
Partner or relative	347/1001 (34.7)
Other[d]	65/392 (22.2)
Test for difference [c]	P<0.001
**Repeated violence[c]**	
No	48/299 (16.1)
Yes	363/983 (36.9)
Test for difference [c]	P<0.001

[a] The prevalence of PTSD associated with characteristics of the assault indicated in the column heading.
[b] Adjusted for current age and number of life stressors.
[c] Adjusted for current age, number of life stressors and years since the worst life stressor (the assault) occurred.
[d] Friend, co-worker, acquaintance, or other.

© 2024 Thordardottir EB et al. *JAMA Network Open.*
