# Supplementary material for: Prevalence of Life Stressors and Posttraumatic Stress Disorder Among Women in Iceland
Source: JAMA Netw Open. 2024 Dec 6;7(12):e2449430. doi: 10.1001/jamanetworkopen.2024.49430 (PMC11624579; doi:10.1001/jamanetworkopen.2024.49430)
Supplement: Supplement 2. — Data Sharing Statement [file jamanetwopen-e2449430-s002.pdf]

Data Sharing Statement

Thordardottir. Prevalence of Life Stressors and Posttraumatic Stress Disorder Among Women in Iceland. *JAMA Netw Open*. Published December 06, 2024.
doi:10.1001/jamanetworkopen.2024.49430

Data
Data available: No

Additional Information
Explanation for why data not available: The data used in this study are compiled in the Stress-And-Gene-Analysis (SAGA) cohort. A detailed description of the study protocol and self-report measures used in this study can be found on the SAGA cohort's website (https://afallasaga.is/english/) and in the supplementary material. According to the ethical approval from the National Bioethics Committee (NBC), individual-level data cannot be made publicly available. The SAGA cohort contains extremely sensitive data, and all use of data is subjected to the approval of NBC (email: vsn@vsn.is). Interested researchers can obtain access to deidentified data by submitting a proposal to the SAGA cohort data management board (email: afallasaga@hi.is) which assists with submitting an amendment to the NBC in obtain data from the study. The corresponding author of the present study submitted a research proposal to the SAGA cohort data management board / the NBC and got access only to deidentified data, that cannot be shared in any way.
